# Factors Associated With Travel Distance in the Receipt of Proton Breast Radiation Therapy

**DOI:** 10.14338/IJPT-22-00018.1

**Published:** 2022-09-20

**Authors:** Sylvia S. Rhodes, Eva Berlin, Nikhil Yegya-Raman, Abigail Doucette, Michelle Gentile, Gary M. Freedman, Neil K. Taunk

**Affiliations:** Department of Radiation Oncology, Perelman School of Medicine at the University of Pennsylvania, Philadelphia, PA, USA

**Keywords:** breast cancer, proton radiation therapy, travel distance

## Abstract

**Introduction:**

Proton radiation therapy (PBT) may reduce cardiac doses in breast cancer treatment. Limited availability of proton facilities could require significant travel distances. This study assessed factors associated with travel distances for breast PBT.

**Materials and Methods:**

Patients receiving breast PBT at the University of Pennsylvania from 2010 to 2021 were identified. Demographic, cancer, and treatment characteristics were summarized. Straight-line travel distances from the department to patients' addresses were calculated using BatchGeo. Median and mean travel distances were reported. Given non-normality of distribution of travel distances, Wilcoxon rank sum or Kruskal-Wallis test was used to determine whether travel distances differed by race, clinical trial participation, disease laterality, recurrence, and prior radiation.

**Results:**

Of 1 male and 284 female patients, 67.8% were White and 21.7% Black. Median travel distance was 13.5 miles with interquartile range of 6.1 to 24.8 miles, and mean travel distance was 13.5 miles with standard deviation of 261.4 miles. 81.1% of patients traveled less than 30 and 6.0% more than 100 miles. Black patients' travel distances were significantly shorter than White patients' and non-Black or non-White patients' travel distances (median = 4.5, 16.5, and 11.3 miles, respectively; *P* < .0001). Patients not on clinical trials traveled more those on clinical trials (median = 14.7 and 10.2 miles, respectively; *P* = .032). There was no difference found between travel distances of patients with left-sided versus right-sided versus bilateral disease (*P* = .175), with versus without recurrent disease (*P* = .057), or with versus without prior radiation (*P* = .23).

**Conclusion:**

This study described travel distances and demographic and clinicopathologic characteristics of patients receiving breast PBT at the University of Pennsylvania. Black patients traveled less than White and non-Black or non-White patients and comprised a small portion of the cohort, suggesting barriers to travel and PBT. Patients did not travel further to receive PBT for left-sided or recurrent disease.

## Introduction

While the clinical efficacy of proton breast radiation therapy (PBT) is comparable to that of photon radiation therapy (RT), RT remains the standard first-line treatment for patients with breast cancer. However, PBT may improve the reduction of cardiac and lung doses due to its unique physical dose distribution that allows for little to no exit dose to structures beyond the target volume [1–4]. Breast PBT may be indicated in situations where RT with 3D or intensity-modulated techniques cannot meet standard benchmarks for safe and acceptable dose constraints. Cases considered for PBT may include regional nodal radiation where photon plans exceed reasonable cardiac and lung doses, re-irradiation, atypical chest wall anatomy—such as severe pectus deformity—special cases of partial breast irradiation, co-morbid cardiopulmonary conditions, and clinical trials [2, 3, 5–7].

PBT is more costly than photon RT, requiring significant capital and personnel investment [8]. In turn, proton centers are generally limited to major academic centers or accessible cities, resulting in potentially significant travel distances [1]. Many patients may find traveling for PBT challenging due to the complex travel and living arrangements required [9]. Travel distance from RT facilities has been demonstrated to be a barrier to recommended care [10]. Despite this, patients may be willing to travel long distances for PBT given the potential clinical and dosimetric advantages not available at their local radiation centers [11–14]. Patients may also be interested in proton clinical trials and may be referred by formal and informal networks for special circumstances, such as re-irradiation. Prior published reports in breast cancer RT have indicated that patients are willing to travel for novel radiation technologies, notably breast intraoperative radiation therapy (IORT)—the same may be true for novel PBT [15].

To our knowledge, there is currently no published information available on factors influencing patient travel to receive breast PBT. This study aimed to examine travel distances for patients receiving breast PBT and to assess clinicopathologic and demographic factors associated with travel distances of those who have received PBT.

## Materials and Methods

### Subject Selection

Patients who received PBT for nonmetastatic breast cancer at the Roberts Proton Therapy Center at the Abramson Cancer Center from its opening on January 25, 2010, to March 16, 2021, were included. Subjects were identified for the study by retrospectively reviewing patient charts at the University of Pennsylvania, Perelman School of Medicine. Our institutional review board approved this retrospective study, complied with the Health Insurance Portability and Accountability Act guidelines, and granted a waiver of consent for the study.

### Data Extraction and Statistical Analysis

Patient demographic data, breast cancer characteristics, and treatment details were extracted from the electronic medical record. Patient demographic characteristics, including age at diagnosis, sex, and self-reported race, and breast cancer characteristics, including tumor laterality and tumor-nodal stage, were summarized. Patients were staged according to the *American Joint Committee on Cancer 7th edition Cancer Staging Guidelines* [16] and patients with recurrent disease were staged as “recurrent.” Treatment details were summarized, including systemic therapy, participation in 1 of our 2 prospective breast PBT clinical trials, modality of radiation, and radiation dosage.

BatchGeo (BatchGeo, LLC, Vancouver, WA) was used to calculate straight-line distances from the Roberts Proton Therapy Center to patients' home addresses. The straight-line distances were used to approximate patients' travel distances to the Center. Mean and median travel distances were reported, and a histogram was used to illustrate the distribution of travel distances. Travel distances were compared by treatment year. Median and mean travel distances for each year were reported along with interquartile range (IQR) and SD. The Wilcoxon rank sum or Kruskal-Wallis test was used to determine whether travel distances differed by race (Black vs White vs other), disease laterality (left vs right vs bilateral), stage (0 vs IA vs IIA vs IIB vs IIIA vs IIIB vs IIIC vs recurrent), recurrence status (yes vs no), chemotherapy use (yes vs no), clinical trial participation (yes vs no), radiation modality (pencil beam scanning vs passive scatter), radiation fractionation (partial vs hypofractionated vs conventional vs hyperfractionated), and prior radiation (yes vs no). These tests were used given non-normality of the distribution of travel distances, as assessed by Shapiro-Wilk test. The χ^2^ tests were done to analyze race and long travel distances. Data were analyzed using Excel 16.51 (Microsoft, Redmond, WA) and SAS OnDemand for Academics.

## Results

### Patient Characteristics

There were 285 patients included as follows: 284 female patients and 1 male patient. Most patients were White (67.7%), and the mean age at diagnosis was 55.6 years. Of patients, 56.1% had left-sided disease, 30.9% had right-sided disease, and 13.0% had bilateral tumors. Overall, 50.9% of patients received chemotherapy; 28.8% received adjuvant chemotherapy and 21.8% received neoadjuvant chemotherapy. Of patients, 36.8% were participating in a clinical trial. Regarding radiation therapy, 21.8% of patients received partial breast irradiation, 8.8% received hypofractionated RT, 58.9% received conventionally fractionated RT, and 10.5% received hyperfractionated RT. Finally, 56.8% of patients received pencil beam scanning (PBS) proton therapy, and 43.2% received scattering proton therapy (SPT). There were 323 tumors identified, and their group, tumor, and node stages are summarized (**Table 1**).

**Table 1.** Demographic, breast cancer, and treatment characteristics.

**Parameter**	**Value**
Demographic	
Total number of patients	285
Gender	
Women, n (%)	284 (99.6)
Men, n (%)	1 (0.4)
Race	
Asian, n (%)	11 (3.9)
Black, n (%)	62 (21.8)
White, n (%)	193 (67.7)
Multiple races, n (%)	6 (2.1)
Other or unknown, n (%)	13 (4.6)
Mean age at diagnosis, y	55.7
Range of age at diagnosis, y	25–83
Insurance type	
Medicaid, n (%)	29 (10.2)
Medicare, n (%)	99 (34.7)
Commercial insurance, n (%)	157 (55.1)
Location	
Urban, n (%)	190 (66.9)
Nonurban, n (%)	94 (33.1)
Breast cancer, n (%)	
Laterality	
Left only	160 (56.1)
Right only	88 (30.9)
Bilateral	37 (13.0)
Stage	
Total tumors	323
Group stage 0	24 (7.4)
Group stage IA	73 (22.6)
Group stage IIA	44 (13.6)
Group stage IIB	44 (13.6)
Group stage IIIA	54 (16.7)
Group stage IIIB	12 (3.7)
Group stage IIIC	23 (7.1)
Recurrent	49 (15.2)
Tumor	
T0	27 (8.4)
T1	117 (36.2)
T2	68 (21.1)
T3	44 (13.6)
T4	18 (5.6)
Recurrent	49 (15.2)
Node	
N0	116 (35.9)
N1	102 (31.6)
N2	33 (10.2)
N3	23 (7.1)
Recurrent	49 (15.2)
Treatment, n (%)	
Chemotherapy	
Total number of patients	145 (50.9)
Adjuvant	82 (28.8)
Neoadjuvant	62 (21.8)
Clinical trial	
Total number of patients	105 (36.8)
RADCOMP [17]	64 (22.5)
Proton APBI	41 (14.4)
Not in clinical trial	180 (63.2)
Radiation modality	
Pencil beam scanning	162 (56.8)
Scattering	123 (43.2)
Radiation fractionation	
Partial	62 (21.8)
Hypofractionated	25 (8.8)
Conventionally fractionated	168 (58.9))
Hyperfractionated	30 (10.5

Abbreviations: RADCOMP, Radiotherapy Comparative Effectiveness; APBI, Accelerated Partial Breast Radiation.

### Travel Distance

The median travel distance to the treatment center was 13.5 miles with an IQR of 6.1 to 24.8 miles, and the mean distance was 64.7 miles with an SD of 261.4 miles. Most (81.1%) travel distances were within 30 miles, and the histogram of travel distances shows a peak at distances between 5 and 10 miles (**Figure**). Six percent of patients traveled more than 100 miles. The median travel distances were fairly consistent when comparing the travel distances by treatment year. The mean travel distances showed more variation, with trends of increasing distances after 2010, peaking in 2014 at 182.8 miles, and declining distances after 2014. The mean travel distances showed more variation (**Table 2**).

**Figure.**
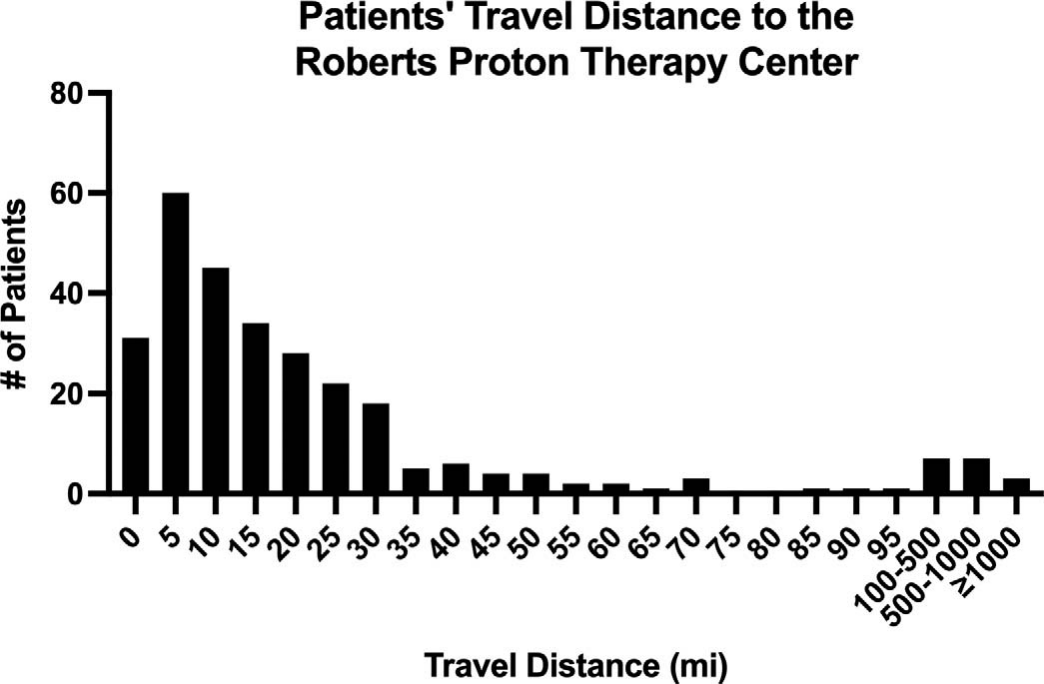
Patients' travel distance to the Roberts Proton Therapy Center.

**Table 2.** Travel distances (miles) by year.

	**2010**	**2012**	**2013**	**2014**	**2015**	**2016**	**2017**	**2018**	**2019**	**2020**	**2021**
n	1	1	8	22	36	35	22	56	58	39	7
Median	22.9	34.4	14.1	13.7	11.3	17.3	24.4	7.5	12.0	16.3	23.9
IQR	n/a	n/a	4.3–32.6	2.5–37.8	6.6–25.0	8.5–24.9	12.9–72.8	4.0–18.5	6.6–24.3	7.7–28.9	4.4–49.2
Mean	22.9	34.4	43.9	182.8	147.0	84.8	107.6	15.2	17.0	21.2	109.3
SD	0.0	0.0	86.6	557.7	410.6	363.9	219.3	34.1	15.3	20.0	240.8

**Abbreviation:** IQR, interquartile range; n/a, not applicable.

Black patients' travel distances (median = 4.5 miles, IQR = 2.83–9.84 miles) were significantly shorter than White patients' travel distances (median = 16.5 miles, IQR = 8.5–27.3 miles) and non-Black or non-White patients' travel distances (median = 11.3 miles, IQR = 10.8–13.5 miles) (*P* < .0001). Non-Black patients were more likely to travel greater than 60 miles when compared with Black patients (*P* = .024). Travel distances of patients not participating in clinical trials (median = 14.7 miles, IQR = 6.9–27.5 miles) were significantly greater than distances of those participating in clinical trials (median = 10.2 miles, IQR = 4.3–21.8 miles) (*P* = .032).

Other analyses observed no other statistically significant differences for travel distances (**Table 3**). Of note, no difference was found between travel distances of patients with left, right, and bilateral disease (*P* = .175), with and without recurrent disease (*P* = .057), with and without chemotherapy (*P* = .057), or with and without prior radiation (*P* = .23). No difference was observed for travel distances of patients with respect to stage (*P* = .75), radiation modality (*P* = .57), or radiation fractionation (*P* = .31).

**Table 3.** Analyses of travel distances.

**Parameter**	**n**	**Median**	**IQR**	**Mean**	**SD**	**Test**	***P*** **value**
Race						Kruskal-Wallis	
Black	62	4.5	2.8–9.8	22.8	111.0	Black vs White vs other	< .0001^a^
White	193	16.5	8.5–27.3	72.7	270.2		
Other	30	11.3	10.8–13.5	87.0	393.1		
Asian	11	27.7	11.5–31.1	217.6	646.9		
Multiple races	6	7.4	4.0–12.8	8.9	6.8		
Other or unknown	13	21.0	4.5–61.9	46.0	68.1		
Age group						Kruskal-Wallis	
< 50	96	13.5	5.2–27.6	59.9	244.0	< 50 vs 50–64 vs > 64	.97
50–64	101	12.9	6.3–24.6	86.9	346.1		
> 64	88	14.0	6.0–24.3	45.0	139.1		
Insurance type						Kruskal-Wallis	
Medicaid	29	4.2	2.5–19.7	14.5	20.2	Medicaid vs Medicare vs commercial	.016^a^
Medicare	99	15.3	6.5–25.3	53.4	156.2		
Commercial	157	13.5	6.6–26.6	81.4	328.9	Medicare vs commercial	.43
Residence type						Kruskal-Wallis	
Urban	190	3.8	2.1–7.8	62.2	350.2	Urban vs nonurban	< .0001^a^
Nonurban	94	20.8	11.3–31.0	68.1	205.3		
Laterality						Kruskal-Wallis	
Left only	160	14.7	6.7–27.9	68.1	313.0	Left vs right vs bilateral	.18
Right only	88	13.7	5.5–24.3	79.6	206.4		
Bilateral	37	9.9	4.5–20.1	15.5	18.5		
Stage						Kruskal-Wallis	
0	24	10.1	4.6–17.3	11.6	8.2	0 vs IA vs IIA vs IIB vs IIIA vs IIIB vs IIIC vs Recurrent	
IA	73	13.0	6.2–29.0	86.8	325.8		.75
IIA	44	7.5	2.8–17.8	30.7	128.7		
IIB	44	14.3	4.2–25.9	71.0	207.4		
IIIA	54	11.7	6.1–21.4	30.2	92.9		
IIIB	12	10.7	6.5–22.4	196.7	621.0		
IIIC	23	23.0	10.4–42.0	35.7	50.0		
Recurrent	49	17.2	8.8–31.3	64.6	292.4		
Recurrence						Wilcoxon rank sum	
Yes	49	17.2	8.8–31.3	64.6	292.4	Yes vs no	.057
No	236	12.6	5.8–24.6	64.9	255.2		
Chemotherapy						Wilcoxon rank sum	
Yes	144	11.3	6.1–22.5	32.1	117.0	Yes vs no	.057
Adjuvant	82	13.5	6.2–22.9	34.8	124.0		
Neoadjuvant	62	10.2	6.0–21.6	28.6	107.9		
No	141	14.7	6.2–31.0	98.3	349.9		
Clinical trial						Wilcoxon rank sum	
Yes	105	10.2	4.3–21.8	44.1	141.7	Yes vs no	.032^a^
No	180	14.7	6.9–27.5	76.9	310.4		
Radiation modality						Wilcoxon rank sum	
Pencil beam Scanning	162	14.2	6.4–25.9	33.9	100.6	PBS vs scattering	.57
Scattering	123	12.8	5.9–24.3	105.7	377.8		
Fractionation						Kruskal-Wallis	
Partial	62	11.4	4.9–28.0	57.8	166.5	Partial vs hypofractionated vs conventional vs Hyperfractionated	.31
Hypofractionated	25	12.2	4.6–22.4	137.0	510.0		
Conventional	168	13.9	6.1–23.9	64.2	259.9		
Hyperfractionated	30	22.0	8.8–31.3	23.2	16.9		
Prior radiation						Wilcoxon rank sum	
Yes	33	16.5	8.8–27.4	20.2	15.4	Yes vs no	.23
No	252	13.5	5.9–24.8	70.7	277.5		

**Abbreviations:** IQR, interquartile range.

a Indicates statistical significance at α = 0.05 for Wilcoxon rank sum or Kruskal-Wallis test.

## Discussion

This study reports the travel distances of patients receiving PBT for nonmetastatic breast cancer at the University of Pennsylvania and how clinicopathologic and demographic characteristics correlate with travel distances. Most patients were local and did not travel far to receive PBT, with a median travel distance of 13.5 miles with IQR of 6.1 to 24.8 miles and a mean of 64.9 miles with SD of 261.4 miles, mainly driven by outliers for average distances. Median travel distances did not change significantly over a decade. Mean travel distances fluctuated, increasing after 2010, peaking in 2014 at 182.8 miles, and declining after 2014. The decline in travel distances after 2014 likely reflected the opening of new PBT centers, and then the COVID-19 pandemic caused a decrease in destination medical care. Black patients traveled significantly less than White and Non-Black or Non-White patients for PBT, and Black patients were less likely to travel more than 60 miles compared with non-Black patients. Patients not participating in clinical trials traveled significantly more than those participating in clinical trials. Likely, patients who did not participate in clinical trials traveled specifically to receive PBT rather than participating in a randomized clinical trial.

Surprisingly, certain patient characteristics considered strong indications for protons did not affect the distance traveled to obtain protons. No difference was found between travel distances of patients with left-sided, right-sided, and bilateral disease, with and without recurrent disease, with and without chemotherapy, or with and without prior radiation. This suggests that patients did not travel farther for PBT due to concerns about increased cardiac toxicity, recurrences, or cumulative radiation received.

Numerous publications on travel distances to receive PBT demonstrate the field's interest in this topic and the magnitude of travel some patients experience to receive treatment. A study conducted at the MD Anderson Cancer Center, where nearly one-third of hospital admissions are from out-of-state patients as it is a destination treatment center, found the median travel distance to receive PBT for medulloblastoma was 1008 miles [11]. Another study at the Washington University School of Medicine showed that cancer patients traveled significantly more for proton than photon therapy, with mean distances of 83.3 and 47.4 miles, respectively [13]. Two separate studies of the National Cancer Database revealed that most patients receiving PBT for prostate cancer traveled more than 100 miles to the treatment facility and that pediatric patients treated with PBT were more likely to travel more than 200 miles than those treated with photon therapy [12, 14]. These results suggest that patients are willing to travel long distances for PBT, particularly in less densely populated states with limited RT access. They also demonstrate the distances traveled by pediatric patients referred to specialty pediatric oncology centers that can offer PBT.

However, reaching PBT treatment facilities requires more than willingness and referrals. Long-distance travel followed by extended stays at far-away centers requires significant time, capital, and social support [9]. Americans living in isolated rural areas have 1-hour longer adjusted travel time to the nearest radiation oncology facility when compared with Americans living in urban areas [18]. A study found that the likelihood of receiving radiotherapy decreases significantly with increasing travel distance to the nearest facility [19]. Older age, being single or widowed, and lower household income were also associated with shorter travel distances, suggesting that those with less resources and social support are less likely to travel greater distances for specialized care [20]. Insurance coverage and income also affect access to PBT. Pediatric patients with private insurance or managed care were more likely to receive PBT than those with Medicaid or no insurance, and prostate cancer patients treated with PBT lived in higher-income counties than patients treated with external beam radiation therapy [12, 14]. The mean age in our study was 55 years, below the age qualifying for Medicare, which is often more permissive to breast PBT than most private insurers. Our study showed that most patients did not travel far for PBT and may provide an additional example of how distance is an obstacle to patients' access to specialized radiation.

Race has also been associated with varying access to RT. Black patients have less access to RT than White patients, independent of but frequently in concert with low socioeconomic status [21]. A recent study on over 5 million cancer patients found that Black patients were less likely to receive PBT than White patients with similar PBT eligibility and availability, and the racial disparity in receipt of PBT was greatest for cancers for which PBT was the recommended therapy [22]. Racial disparities were also reflected in our analysis. Most of the patients in our PBT population were local, and Philadelphia's population comprises 40.7% White and 42.1% Black residents. However, this relatively even ratio was not consistent with the demographics of our PBT population, which was 67.8% White and only 21.7% Black. Black patients were also less likely to travel more than 60 miles compared with non-Black patients. This may be due to a combination of unequal access to PBT among Black patients and the demographics in the suburban areas surrounding Philadelphia, which are mostly White. Understanding racial disparities and the barriers that prevent Black patients from receiving equitable radiation care warrants further study.

Several limitations should be noted. The study is based in a single center and retrospective, so results may not be generalizable to cancer patients in other clinical settings. Patients were not randomized to receive PBT or another therapy. There is no direct comparison to patients who received photon therapy for breast cancer, as photon patients are generally referred directly to the University of Pennsylvania's regional treatment sites. Although most patients traveled short distances to receive treatment, a small number of patients traveled particularly great distances, hundreds or thousands of miles, which greatly elevated mean travel distances and SDs.

## Conclusion

Overall, this study summarized and described travel distances, demographics, and clinicopathologic characteristics of patients receiving breast PBT at the University of Pennsylvania. We found that Black patients traveled lesser distances than non-Black patients and comprised a small portion of the cohort, suggesting barriers to travel and limited access to PBT. Racial disparities and the barriers to equitable radiation care for the Black patient experience need to be further studied. Patients not participating in clinical trials traveled significantly more than those participating in clinical trials, suggesting that patients traveled specifically for PBT but not randomized clinical trials. Patients did not appear to travel longer distances to receive PBT for left-sided disease, recurrent disease, or prior radiation. This study adds to the body of literature on the implications of distance in PBT treatment and highlights the importance of access to PBT for the appropriate patient.
